# Supplementary material for: Derivation and external validation of clinical prediction rules identifying children at risk of linear growth faltering
Source: eLife. 2023 Jan 6;12:e78491. doi: 10.7554/eLife.78491 (PMC9833824; doi:10.7554/eLife.78491)
Supplement: Supplementary file 1. [file elife-78491-supp1.pdf]

Derivation and external validation of clinical prediction rules identifying children at risk of linear growth faltering

Sharia M. Ahmed[1], Ben J. Brintz[2], Patricia B Pavlinac[3], Lubaba Shahrin[4], Sayeeda Huq[4], Adam C. Levine[5], Eric J. Nelson[6], James A Platts-Mills[7], Karen L Kotloff[8], Daniel T Leung[1]

Affiliations:
1. Division of Infectious Diseases, University of Utah School of Medicine, Salt Lake City, UT, USA
2. Division of Epidemiology, University of Utah School of Medicine, Salt Lake City, UT, USA
3. Department of Global Health, Global Center for Integrated Health of Women, Adolescents and Children (Global WACh), University of Washington, Seattle, WA, USA
4. International Centre for Diarrhoeal Disease Research, Bangladesh, Dhaka, Bangladesh
5. Department of Emergency Medicine, Warren Alpert Medical School of Brown University, Providence, RI, USA
6. Department of Pediatrics and Environmental and Global Health, Emerging Pathogens Institute, University of Florida, Gainesville, FL, USA
7. Division of Infectious Diseases and International Health, University of Virginia, Charlottesville, VA, USA
8. Department of Pediatrics, Center for Vaccine Development, University of Maryland School of Medicine, Baltimore, MD, USA

SUPPLEMENT

Supplementary file 1A: Full list of considered predictor variables

GEMS cases	GEMS controls	MAL-ED
Study site (site)<br>Child sex (f3_gender)<br>Loss of skin turgor (f3_drh_turgor)<br>Intravenous rehydration (f3_drh_iv)<br>Hospitalized (f3_drh_hosp)<br>Your relationship to the child (f4a_relationship)<br>Where child's father lives (f4a_dad_live)<br>Primary caregiver's max school (f4a_prim_schl)<br>People living in house 6 months (f4a_ppl_house)<br>Children under 60 months in the house (f4a_yng_children)<br>How many rooms used for sleeping (f4a_slp_rooms)<br>Predominant floor (f4a_floor)<br>Electricity (f4a_house_elec)<br>Bicycle/rickshaw (f4a_house_bike)<br>Telephone (f4a_house_phone)<br>Television (f4a_house_tele)<br>Car/truck (f4a_house_car)<br>Animal-drawn cart (f4a_house_cart)<br>Motorcycle/scooter (f4a_house_scoot)<br>Refrigerator (f4a_house_fridge)<br>Agricultural land (f4a_house_agland)<br>Radio (f4a_house_radio)<br>Boat with motor (f4a_house_boat)<br>None of the above assets (f4a_house_none)<br>Electricity (f4a_fuel_elec)<br>Biogas (f4a_fuel_biogas)<br>Straw/shrubs/grass (f4a_fuel_grass)<br>Liquid propane gas (f4a_fuel_propane)<br>Coal/lignite (f4a_fuel_coal)<br>Animal dung (f4a_fuel_dung)<br>Natural gas (f4a_fuel_natgas)<br>Charcoal (f4a_fuel_charcoal)	Study site (site)<br>Your relationship to the child (f7_relation)<br>Where child's father lives (f7_dad_live)<br>Primary caregiver's max school (f7_prim_schl)<br>People living in house 6 months (f7_ppl_house)<br>Children under 60 months in the house (f7_yng_childrn)<br>How many rooms used for sleeping (f7_slp_rooms)<br>Predominant floor (f7_floor)<br>Electricity (f7_house_elec)<br>Bicycle/rickshaw (f7_house_bike)<br>Telephone (f7_house_phone)<br>Television (f7_house_tele)<br>Car/track (f7_house_car)<br>Animal-drawn cart (f7_house_cart)<br>Motorcycle/scooter (f7_house_scoot)<br>Refrigerator (f7_house_fridge)<br>Agricultural land (f7_house_agland)<br>Radio (f7_house_radio)<br>Moat with motor (f7_house_boat)<br>None of the above assets (f7_house_none)<br>Electricity (f7_fuel_elec)<br>Biogas (f7_fuel_biogas)<br>Straw/shrubs/grass (f7_fuel_grass)<br>Liquid propane gas (f7_fuel_propane)<br>Coal/lignite (f7_fuel_coal)<br>Animal dung (f7_fuel_dung)<br>Natural gas (f7_fuel_natgas)<br>Charcoal (f7_fuel_charcoal)<br>Agricultural crop residue (f7_fuel_crop)<br>Kerosene (f7_fuel_kero)<br>Wood (f7_fuel_wood)<br>Other fuel (f7_fuel_other)<br>Goat (f7_ani_goat)<br>Sheep (f7_ani_sheep)	Cumulative diarrheal episode count (diar_epi_ct)<br>HAZ measurement no more than 31 days before onset of diarrhea (HAZ_1)<br>Diarrheal episode duration (days) (diar_dur)<br>Cumulative days within diarrheal episodes (diar_days_sum)<br>Dairrhea duration categorization (diar_dur_cat)<br>Max loose stools at episode (loose_stool_max)<br>Blood in stool at episode (blood)<br>Days with vomiting at episode (vomit_dur)<br>Days with decreased appetite at episode (app_dec_dur)<br>Max dehydration categorization (dehyd_max_cat)<br>Fever at episode categorization (fev_bin)<br>Cumulative days in this ALRI episode (ALRI_dur)<br>Age (days) (age)<br>Total days of breastfeeding (breast_totdays**)<br>Days since last diarrheal episode (diar_days_since)<br>ORT administered, caregiver report (ORT_caregiver)<br>Hospitalized (hosp)<br>Indrawing, fieldworker assessment (indraw_any)<br>Caregiver reported sleepiness (sleepy_any)<br>Caregiver reported difficult to awaken (unawake_any)<br>Caregiver reported use of antibiotics (abx_any)<br>Caregiver reported use of ORT (ORT_any)<br>If hospitalized that diarrhea episode (hosp_any)<br>First day of the diarrhea episode (first_diar_day)

Agricultural crop residue (f4a_fuel_crop)<br>Kerosene (f4a_fuel_kero)<br>Wood (f4a_fuel_wood)<br>Other fuel (f4a_fuel_other)<br>Goat (f4a_ani_goat)<br>Sheep (f4a_ani_sheep)<br>Dog (f4a_ani_dog)<br>Cat (f4a_ani_cat)<br>Cow (f4a_ani_cow)<br>Rodents (f4a_ani_rodents)<br>Fowl (f4a_ani_fowl)<br>Other animal (f4a_ani_other)<br>No animals (f4a_ani_no)<br>Water piped to house (f4a_water_house)<br>Covered well in house/yard (f4a_water_covwell)<br>Water piped into yard (f4a_water_yard)<br>Covered public well (f4a_water_covpwell)<br>Public tap (f4a_water_pubtap)<br>Protected spring (f4a_water_prospring)<br>Open well in house/yard (f4a_water_well)<br>Unprotected spring (f4a_water_unspring)<br>Open public well (f4a_water_pubwell)<br>River/stream (f4a_water_river)<br>Pond/lake (f4a_water_pond)<br>Deep tube well (f4a_water_deepwell)<br>Rainwater (f4a_water_rain)<br>Shallow tube well (f4a_water_shallwell)<br>Bought water (f4a_water_bought)<br>Other water source (f4a_water_othr)<br>Bore hole (f4a_water_bore)<br>Main source of drinking water (f4a_ms_water*)<br>How often is water available (f4a_water_avail)<br>Did you give the child stored water (f4a_store_water)<br>Do you usually treat drinking water? (f4a_trt_water)	Dog (f7_ani_dog)<br>Cat (f7_ani_cat)<br>Cow (f7_ani_cow)<br>Rodents (f7_ani_rodents)<br>Fowl (f7_ani_fowl)<br>Other animals (f7_ani_other)<br>No animals f7_ani_no<br>Water piped to house (f7_water_house)<br>Covered well in house/yard (f7_water_covwell)<br>Water piped into yard (f7_water_yard)<br>Covered public well (f7_water_covpwell)<br>Public tap (f7_water_pubtap)<br>Protected spring (f7_water_prospring)<br>Open well in house/yard (f7_water_well)<br>Unprotected spring (f7_water_unspring)<br>Open public well (f7_water_pubwell)<br>River/stream (f7_water_river)<br>Pond/lake (f7_water_pond)<br>Deep tube well (f7_water_deepwell)<br>Rainwater (f7_water_rain)<br>Shallow tube well (f7_water_shallwell)<br>Bought water (f7_water_bought)<br>Other water source (f7_water_othr)<br>Bore hole (f7_water_bore)<br>Main source of drinking water (f7_ms_water*)<br>How often is water available (f7_water_avail)<br>Did you give the child stored water? (f7_store_water)<br>Do you usually treat drinking water? (f7_trt_water) Do you usually treat drinking water? (f7_trt_method)<br>How are child's feces disposed (f7_disp_feces)<br>Facility use to dispose of feces (f7_fac_waste)	Breastfed within first 24hr of birth (breast_24)<br>Time between birth and first breastfeeding (time_to_breast)<br>Fed colostrum (colostrum)<br>Prelacteal feeding (prelacteal)<br>Sex (sex)<br>Total days in all diarrheal episodes (tot_diar)<br>Drinking water source (water_source)<br>Persons sleeping in dwelling (ppl_slp)<br>Mean people per room (mean_ppl)<br>Improved/unimproved sanitation (sani_score)<br>Improve/unimproved drinking water (water_score)<br>Household has a bed (bed)<br>Household has a television (tv)<br>Household has a refrigerator (fridge)<br>Household has a table (table)<br>Household has a chai (chair)<br>Main material of roof (roof)<br>Main material of floor (floor)<br>Main material of walls (wall)<br>Household has a bank account (bank)<br>Kitchen located in a separate room (kitchen)<br>Years of formal education mother received (edu)<br>Mother ever attended formal schooling (edu2)<br>Total number of rooms in house (rm_ct)<br>Noniles of average monthly household income in USD (income_score)<br>Fewer than 2 people per room (two_ppl_rm)<br>Toilet/latrine has concrete floor (sani_concrete)<br>Toilet/latrine type (sani_type)<br>Country (country)

Usual treatment method (f4a_trt_method)<br>How are child’s feces disposed (f4a_disp_feces)<br>Facility used to dispose of feces (f4a_fac_waste)<br>How many households share facility? (f4a_share_fac)<br>Wash hands before eating? (f4a_wash_eat)<br>Wash hands before cooking (f4a_wash_cook)<br>Wash hands before you nurse? (f4a_wash_nurse)<br>Wash hands after you defecate (f4a_wash_def)<br>Wash hands after handling animals (f4a_wash_animal)<br>Wash hands after cleaning a child (f4a_wash_child)<br>Wash hands other times (f4a_wash_othr)<br>What do you use to wash your hands? (f4a_wash_use)<br>Is the child currently breastfed? (f4a_breastfed)<br>How long as this diarrhea episode lasted (days)? (f4a_drh_days)<br>Maximum number of loose stools (f4a_max_stools)<br>Blood in stools (f4a_drh_blood)<br>Vomiting 3 or more times per day (f4a_drh_vomit)<br>Very thirsty (f4a_drh_thirst)<br>Drank much less than usual (f4a_drh_lessdrink)<br>Belly pain (f4a_drh_bellypain)<br>Irritable or restless (f4a_drh_restless)<br>Decreased activity or lethargy (f4a_drh_lethrgy)<br>Loss of consciousness (f4a_drh_consc)<br>Rectal straining (f4a_drh_strain)<br>Rectal prolapse (f4a_drh_prolapse)<br>Cough (f4a_drh_cough)<br>Convulsions (f4a_drh_conv)<br>Very thirsty (f4a_cur_thirsty)<br>Wrinkled skin (f4a_cur_skin)	Wash hands before eating? (f7_wash_eat)<br>Wash hands before cooking? (f7_wash_cook)<br>Wash hands before you nurse (f7_wash_nurse)<br>Wash hands after you defecate? (f7_wash_def)<br>Wash hands after handling animals? (f7_wash_animal)<br>Wash hands after cleaning child? (f7_wash_child)<br>Wash hands other times? (f7_wash_othr)<br>What do you use to wash your hands? (f7_wash_use)<br>Is the child currently breastfeeding? (f7_breastfed)<br>Seek outside care? (f7_seekcare)<br>Length/height-for-age z-score (f7_haz)<br>Axillary temperature (f7_temp)<br>Calculated respiratory rate (f7_resp)<br>Bipedal edema (f7_bipedal)<br>Abnormal hair (f7_abn_hair)<br>Undernutrition (f7_under_nutr)<br>Skin as ‘flaky paint’ appearance (f7_skin_flaky)<br>Child age (months) (base_age)	

Irritable or restless (f4a_cur_restless)<br>Dry mouth (f4a_cur_drymouth)<br>Fast breathing (f4a_cur_fastbreath)<br>ORALITE or ORS (f4a_hometrt_ors)<br>Homemade fluid (f4a_hometrt_maize)<br>Special mile or infant formula (f4a_hometrt_milk)<br>Home remedy/herbal medication (f4a_hometrt_herb)<br>Zinc (f4a_hometrt_zinc)<br>No special remedies given (f4a_hometrt_none)<br>Any other liquids (f4a_hometrt_othrliq)<br>Antibiotics (f4a_hometrt_ab)<br>Other treatment (f4a_hometrt_othr1)<br>Other treatment (f4a_hometrt_othr2)<br>How much offered to drink (f4a_offr_drink)<br>Seek outside care (f4a_seek_outside)<br>Pharmacy (f4a_seek_pharm)<br>Friend/relative (f4a_seek_friend)<br>Traditional healer (f4a_seek_healer)<br>Unlicensed practitioner (f4a_seek_doc)<br>Licensed practitioner (f4a_seek_privdoc)<br>Bought a remedy (f4a_seek_remdy)<br>Other hospital/center (f4a_seek_other)<br>Length/height-for-age z-score (f4b_haz)<br>Axillary temperature (f4b_temp)<br>Respiratory rate per minute (f4b_resp)<br>Chest indrawing (f4b_chest_indrw)<br>Eyes (f4b_eyes)<br>Mouth (f4b_mouth)<br>Skin pinch (f4b_skin)<br>Mental status (f4b_mental)<br>Rectal prolapse (f4b_rectal)		

Bipedal edema (f4b_bipedal)<br>Abnormal hair (f4b_abn_hair)<br>Undernutrition (f4b_under_nutr)<br>Skin as 'flaky paint' appearance (f4b_skin_flaky)<br>Staff observed a stool sample (f4b_observe_stool)<br>Nature of the stool (f4b_nature_stool)<br>Receive rehydration here (f4b_recommend)<br>Child was admitted to hospital (f4b_admit)<br>Child age (months) (base_age)		

*f4a_ms_water and f7_ms_water were recategorized into the following: surface, other unimproved, other improved, piped, other(1, 2)
**breast_totdays is calculated from the cumulative number of days of each of the following types of breastfeeding, combined using the listed formula: exclusive, predominant, partial, and none; breast_totdays=(breast_excl*1)+(breast_predom*0.75)+(breast_part*0.5)+(breast_not*0))

Supplementary file 1B: Total sample size and growth faltering in GEMS by site

N's	The Gambia	Mali	Mozambique	Kenya	India	Bangladesh	Pakistan
Median HAZ at enrollment	-1.2	-0.9	-1.2	-1.3	-1.3	-1.2	-1.9
≥0.5 decrease in HAZ	251 (31.9%)	255 (14.9%)	145 (34.5%)	296 (28.4%)	269 (18.5%)	304 (23.1%)	224 (24.9%)
≥1.0 decrease in HAZ	48 (6.1%)	51 (3.0%)	42 (10.0%)	76 (7.3%)	55 (3.8%)	38 (2.9%)	47 (5.2%)
Total	788	1715	420	1042	1457	1316	901

Supplementary file 1C: Total Sample size and growth faltering in GEMS by age

N's	0-11mo	12-23mo	24-59mo
≥0.5 decrease in HAZ	1077 (33.7%)	584 (22.3%)	83 (4.6%)
≥1.0 decrease in HAZ	293 (9.2%)	64 (2.4%)	0 (0%)
Total	3196	2622	1821

Supplementary file 1D: Variable importance ordering, cross-validated average AUC, odds ratios, and 95% confidence intervals for logistic regression models predicting growth faltering (≥0.5 decrease in HAZ) in children 0-59mo in LMICs in GEMS and MAL-ED ranked from most to less predictive (highest to lower variance reduction)

GEMS 0-59mo		MAL-ED 0-23mo	
AUC (95% CI): 0.72 (0.719, 0.724)		AUC (95% CI): 0.68 (0.67, 0.69)	
Variables	OR (95% CI)	Variables	OR (95% CI)
Age (months)	0.92 (0.91, 0.93)	HAZ	1.35 (1.17, 1.55)
HAZ	1.30 (1.24, 1.36)	Age (days)	1.00 (1.00, 1.00)
Respiratory rate	1.00 (0.99, 1.00)	Total days breastfed	1.00 (1.00, 1.01)
Temperature	1.28 (1.21, 1.36)	Tot. days with diarrhea during study (tot_diar)	0.98 (0.97, 1.00)
Num. ppl living in household	1.00 (0.99, 1.01)	Mean num. people per room	1.05 (0.94, 1.16)
Num. ppl sleeping in household	1.00 (0.98, 1.03)	Num. days of diarrhea in lifetime at presentation (diar_days_sum)	1.02 (0.99, 1.04)
Num. days of diarrhea at presentation	0.98 (0.93, 1.02)	Maternal education (years)	0.95 (0.91, 1.00)
Num. other households share fecal waste disposal facility	0.99 (0.97, 1.00)	Num. days since last diarrhea episode (diar_days_since)	1.00 (1.00, 1.00)
Currently breastfeeding	0.85 (0.74, 0.97)	Num. ppl. sleep in dwelling	1.07 (1.01, 1.12)
Num. children <60months live in household	0.99 (0.95, 1.03)	Max. num. loose stools per day in this diarrhea episode	0.93 (0.86, 1.00)
2-variable model fit to 0-23mo			
AUC (95% CI): 0.63 (0.62, 0.65)			
Age (months)	0.95 (0.94, 0.96)		
HAZ	1.31 (1.25, 1.38)		

Supplementary file 1E: **GROWTH FALTERING:** Variable importance ordering and cross-validated average overall AUC and AUC by patient subset and 95% confidence intervals for a 2 (plain text), 5 (bold), and 10 (italicized) variable logistic regression model for predicting growth faltering in children derived in GEMS and MAL-ED data (≥0.5 decrease in HAZ)

Data	GEMS	GEMS	MAL-ED	GEMS	MAL-ED	GEMS	MAL-ED	GEMS	GEMS
Patient Subset	0-59mo (main text model)	0-11mo	0-11mo	12-23mo	12-23mo	0-23mo (for external validation)	0-23mo	24-59mo	0-59mo outcome is any stunting at follow-up
AUCs	**0.72 (0.72, 0.72)**	**0.60 (0.59, 0.60)**	**0.60 (0.60, 0.61)**	**0.66 (0.65, 0.66)**	**0.65 (0.64, 0.66)**	**0.64 (0.63, 0.64)**	**0.62 (0.61, 0.63)**	**0.78 (0.77, 0.79)**	**0.96 (0.96, 0.96)**
	0.72 (0.71, 0.73)	*0.60 (0.59, 0.60)*	*0.63 (0.62, 0.63)*	*0.70 (0.69, 0.70)*	*0.64 (0.63, 0.65)*	*0.64 (0.64, 0.64)*	*0.63 (0.62, 0.64)*	*0.76 (0.75, 0.77)*	*0.96 (0.96, 0.97)*
1	Age (months)	HAZ	HAZ	HAZ	Age (days)	HAZ	HAZ	Temp	HAZ
2	HAZ	Temp	Total days breastfeeding	Respiratory rate	HAZ	Age (months)	Age (days)	Respiratory rate	Age (months)
3	Respirator rate	Respiratory rate	Age (days)	Temp	Total days breastfeeding	Temperature	Total days breastfeeding	Age (months)	Wasted / very thin
4	Temperature	Num. people living in household	Tot. days with diarrhea during study	Age (months)	Tot. days with diarrhea during study	Respiratory rate	Tot. days with diarrhea during study	HAZ	Respiratory rate
5	Num. people living in household	Age (months)	Mean num. of people per room	Num. people living in household	Num. days since last diarrhea episode	Num. people living in household	Mean num. people per room	Num. people living in household	Temperature
6	Num. rooms used for sleeping	Num. other households share latrine	Num. days of diarrhea in lifetime at presentation	Num. rooms used for sleeping	Num. days of diarrhea in lifetime at presentation	Num. rooms used for sleeping	Num. days of diarrhea in lifetime at presentation	Num. other households that share same fecal waste facility	Num. people living in household

7	Num. days of diarrhea at presentation	Num. days of diarrhea at presentation	Num. ppl. sleep in dwelling	Received recommended rehydration at health center	Mean num. people per room	Num. days of diarrhea at presentation	Num. ppl. Sleep in dwelling	Num. days of diarrhea at presentation	Site
8	Num. other households that share same fecal waste facility	Num. rooms used for sleeping	Max num. loose stools per day in this diarrhea episode	Num. days of diarrhea at presentation	Maternal education (years)	Num. other households that share same fecal waste facility	Maternal education (years)	Num. rooms used for sleeping	Num. other households that share same fecal waste facility
9	Breastfed	Num. children <60months live in household	Maternal education (years)	Num. other households that share same fecal waste facility	Num. ppl sleep in dwelling	Num. children <60 months live in household	Num. days since last diarrhea episode	Num. children <60months live in household	Num. rooms used for sleeping
10	Num. children <60months live in household	Caregiver education	Num. days since last diarrhea episode	Num. children <60months live in household	Num. of diarrhea episodes in lifetime	Caregiver education	Avg. monthly household income in USD, noniles	Type of fecal waste facility used by household	Num. days of diarrhea at presentation

Data	GEMS	GEMS	GEMS	GEMS	GEMS	GEMS
Patient Subset	0-59mo (main text model) only HAZ considered	Only MUAC considered	HAZ + MUAC considered	main + month of diarrhea	Abx (b/f, during, rx, ever)	≥1.0 ΔHAZ in 0-59mo
AUCs	**0.72 (0.72, 0.72)**	**0.70 (0.70, 0.70)**	**0.72 (0.72, 0.73)**	**0.72 (0.72, 0.72)**	**0.72 (0.72, 0.72)**	**0.80 (0.79, 0.80)**
	0.72 (0.71, 0.73)	*0.70 (0.70, 0.70)*	*0.72 (0.72, 0.73)*	*0.72 (0.72, 0.72)*	*0.72 (0.72, 0.72)*	*0.80 (0.79, 0.80)*
1	Age (months)	Age (months)	Age (months)	Age (months)	Age (months)	Age (months)
2	HAZ	MUAC	HAZ	HAZ	HAZ	HAZ
3	Respirator rate	Respiratory rate	MUAC	Respiratory rate	Respiratory rate	Respiratory rate
4	Temperature	Temperature	Respiratory rate	Temperature	Temperature	Temperature
5	Num. people living in household	Num. people living in household	Temperature	Num. people living in household	Num. people living in household	Num. people living in household
6	Num. rooms used for sleeping	Num. days of diarrhea at presentation	Num. people living in household	Month	Num. rooms used for sleeping	Num. other households that share same fecal waste facility
7	Num. days of diarrhea at presentation	Num. rooms used for sleeping	Num. rooms used for sleeping	Num. rooms used for sleeping	Num. days of diarrhea at presentation	Num. rooms used for sleeping
8	Num. other households that share same fecal waste facility	Num. other households that share same fecal waste facility	Num. days of diarrhea at presentation	Num. days of diarrhea at presentation	Num. other households that share same fecal waste facility	Num. days of diarrhea at presentation
9	Breastfed	Breastfed	Num. other households that share same fecal waste facility	Num. other households that share same fecal waste facility	Breastfed	Num. children <60months live in household
10	Num. children <60months live in household	Num. children <60months live in household	Breastfed	Breastfed	Num. children <60months live in household	Breastfed
Rank of additional variable	N/A	N/A	N/A	6th	62nd (rx)<br>71st (during)<br>78th (ever)<br>84th (before)	N/A

Data Attributable fraction cutoff considered	GEMS	0.3	0.3	0.3	0.3	0.5	0.5	0.5	0.5	0.7	0.7	0.7	0.7
Patient Subset	0-59mo (main text model)	main + Y/N Shigella*	main + Y/N crypto*	Main + Y/N Shigella + Y/N crypto*	main + Y/N any viral**	main + Y/N Shigella*	main + Y/N crypto*	Main + Y/N Shigella + Y/N crypto*	main + Y/N any viral**	main + Y/N Shigella*	main + Y/N crypto*	Main + Y/N Shigella + Y/N crypto*	main + Y/N any viral**
AUCs	**0.72 (0.72, 0.72)**	**0.73 (0.73, 0.74)**	**0.73 (0.73, 0.74)**	**0.73 (0.73, 0.74)**	**0.73 (0.73, 0.74)**	**0.73 (0.73, 0.74)**	**0.73 (0.73, 0.73)**	**0.73 (0.73, 0.74)**	**0.73 (0.73, 0.74)**	**0.73 (0.73, 0.74)**	**0.73 (0.73, 0.74)**	**0.73 (0.73, 0.74)**	**0.73 (0.73, 0.74)**
	0.72 (0.71, 0.73)	*0.73 (0.73, 0.74)*	*0.73 (0.73, 0.73)*	*0.73 (0.73, 0.74)*	*0.73 (0.73, 0.73)*	*0.73 (0.73, 0.73)*	*0.73, (0.73, 0.73)*	*0.73 (0.73, 0.74)*	*0.73 (0.73, 0.74)*	*0.73 (0.73, 0.73)*	*0.73, (0.73, 0.74)*	*0.73 (0.73, 0.73)*	*0.73 (0.73, 0.73)*
1	Age (months)	Age (months)	Age (months)	Age (months)	Age (months)	Age (months)	Age (months)	Age (months)	Age (months)	Age (months)	Age (months)	Age (months)	Age (months)
2	HAZ	HAZ	HAZ	HAZ	HAZ	HAZ	HAZ	HAZ	HAZ	HAZ	HAZ	HAZ	HAZ
3	Respirator rate	Resp rate	Resp rate	Resp rate	Resp rate	Resp rate	Resp rate	Resp rate	Resp rate	Resp rate	Resp rate	Resp rate	Resp rate
4	Temperature	Temperature	Temperature	Temperature	Temperature	Temperature	Temperature	Temperature	Temperature	Temperature	Temperature	Temperature	Temperature
5	Num. people living in household	Num. people living in household	Num. people living in household	Num. people living in household	Num. people living in household	Num. people living in household	Num. people living in household	Num. people living in household	Num. people living in household	Num. people living in household	Num. people living in household	Num. people living in household	Num. people living in household
6	Num. rooms used for sleeping	Num. days of diarrhea at	Num. days of diarrhea at	Num. days of diarrhea at	Num. days of diarrhea at	Num. days of diarrhea at	Num. days of diarrhea at	Breastfed	Num. days of diarrhea at	Num. days of diarrhea at	Num. days of diarrhea at	Num. days of diarrhea at	Breastfed

		presentation	presentation	presentation	presentation	presentation	presentation		presentation	presentation	presentation	presentation	
7	Num. days of diarrhea at presentation	Num. rooms used for sleeping	Breastfed	Num. rooms used for sleeping	Num. rooms used for sleeping	Breastfed	Breastfed	Num. days of diarrhea at presentation	Breastfed	Breastfed	Breastfed	Num. rooms used for sleeping	Num. days of diarrhea at presentation
8	Num. other households that share same fecal waste facility	Breastfed	Num. rooms used for sleeping	Breastfed	Breastfed	Num. rooms used for sleeping	Num. rooms used for sleeping	Num. rooms used for sleeping	Num. rooms used for sleeping	Num. rooms used for sleeping	Num. rooms used for sleeping	Breastfed	Num. rooms used for sleeping
9	Breastfed	Num. other households that share same fecal waste facility	Num. other households that share same fecal waste facility	Num. other households that share same fecal waste facility	Num. other households that share same fecal waste facility	Num. other households that share same fecal waste facility	Num. other households that share same fecal waste facility	Num. other households that share same fecal waste facility	Num. other households that share same fecal waste facility	Num. other households that share same fecal waste facility	Num. other households that share same fecal waste facility	Num. other households that share same fecal waste facility	Num. other households that share same fecal waste facility
10	Num. children <60months live in household	Num. children <60months live in household	Num. children <60months live in household	Num. children <60months live in household	Num. children <60months live in household	Num. children <60months live in household	Num. children <60months live in household	Num. children <60months live in household	Num. children <60months live in household	Num. children <60months live in household	Num. children <60months live in household	Num. children <60months live in household	Num. children <60months live in household
Rank of addition	n/a	33rd	18th	19th (crypto)	22nd	49th	20th	22nd (crypto)	26th	60th	56th	52nd (crypto)	26th

al variable				30th (shigella)				48th (shigella)				62nd (shigella)	

*n=4277 due to missing etiology data

**viral etiology include astrovirus, norovirus GII, rotavirus, sapovirus, and adenovirus, n=4277 due to missing etiology data

	The Gambia	Mali	Mozambique	Kenya	India	Bangladesh	Pakistan	Fit in data from Africa	Fit in data from Asia
AUCs			sample size too small					0.72 (0.72, 0.73)	0.70 (0.70, 0.70)
	**0.67 (0.66, 0.67)**	**0.82 (0.82, 0.83)**		**0.71 (0.70, 0.72)**	**0.76 (0.75, 0.76)**	**0.71 (0.71, 0.72)**	**0.68 (0.67, 0.69)**	**0.73 (0.72, 0.73)**	**0.72 (0.71, 0.72)**
	0.69 (0.68, 0.70)	*0.82 (0.81, 0.82)*		*0.72 (0.71, 0.72)*	*0.77 (0.76, 0.78)*	*0.70 (0.70, 0.71)*	*0.68 (0.67, 0.69)*	*0.73 (0.72, 0.73)*	*0.72 (0.72, 0.72)*
1	HAZ	Age		Age	HAZ	Age	Age	Age	Age
2	Age	HAZ		HAZ	Age	HAZ	HAZ	HAZ	HAZ
3	Respiratory rate	Respiratory rate		Respiratory rate	Temperature	Respiratory rate	Temperature	Respiratory rate	Respiratory rate
4	Num. people living in household	Temperature		Temperature	Respiratory rate	Temperature	Respiratory rate	Temperature	Temperature
5	Temperature	Breastfed		Breastfed	Num. other households that share same fecal waste facility	Num. people living in household	Num. people living in household	Num. people living in household	Num. people living in household
6	Num. rooms used for sleeping	Num. people living in household		Num. days of diarrhea at presentation	Num. people living in household	Num. days of diarrhea at presentation	Num. days of diarrhea at presentation	Breastfed	Num. other households that share same fecal waste facility
7	Num. children <60months live in household	Num. rooms used for sleeping		Num. people living in household	Num. days of diarrhea at presentation	Type of fecal waste facility used by household	Num. children <60months live in household	Num. rooms used for sleeping	Num. days of diarrhea at presentation
8	Num. days of diarrhea	Num. children <60months		Num. other households that share	Caregiver education	Num. rooms used for sleeping	Num. rooms used for sleeping	Num. days of diarrhea	Num. rooms used for sleeping

	at presentation	live in household		same fecal waste facility				at presentation	
9	How much offering child to drink	Num. other households that share same fecal waste facility		How much offering child to drink	Num. rooms used for sleeping	Num. other households that share same fecal waste facility	Caregiver education	Num. children <60months live in household	Caregiver education
10	How often main water source available	Num. days of diarrhea at presentation		Num. children <60months live in household	How much offering child to drink	Caregiver education	Child receive recommended rehydration	Num. other households that share same fecal waste facility	Type of fecal waste facility used by household
								2-variable CPR performance in data from Asia	2-variable CPR performance in data from Africa
								0.70 (0.68, 0.72)	0.72 (0.71, 0.74)

REFERENCES

1. Bank TW. Metadata Glossary: Improved water source (% of population with access).
2. Progress on household drinking water, sanitation and hygiene 2000-2020: Five years into the SDGs. Geneva: World Health Organization (WHO) and the United Nations Children's Fund (UNICEF); 2021.
